# Moving to Medical Treatment for COVID-19 Influence on Pediatric Appendicitis: A Meta-Analysis

**DOI:** 10.7759/cureus.32601

**Published:** 2022-12-16

**Authors:** Salman M Ghazwani

**Affiliations:** 1 Surgery, Jazan University, Jizan, SAU

**Keywords:** antibiotics, laproscopic appendectomy, appendectomy, meta analysis, peditric acute appendicitis

## Abstract

Acute appendicitis (AA) is cited as the leading cause of surgical acute abdomen in pediatrics and the most frequent urgent surgical pathology worldwide. For a long time, surgical appendectomy has been effectively used as the first-line treatment for AA. Other conservative management practices, such as the use of antibiotics, have been applied in the treatment of appendicitis. COVID-19 has had a significant impact on the surgical treatment strategies of AA in pediatrics, with many pediatric surgeons having to shift from upfront surgical appendectomy to conservative management involving the use of antibiotics as a treatment strategy. This meta-analysis compares the outcomes between appendectomy and conservative therapy in the management of AA during COVID-19 in pediatrics. Twenty-one articles fully met the inclusion criteria. Articles that were published more than five years ago were excluded from the analysis. Also, articles that included studies on the adult population were excluded. Results from various retrospective studies, prospective clinical controlled trials, correlational studies, and randomized clinical trials were analyzed. This study reveals that the use of antibiotics has been demonstrated to be safe and effective in the treatment of uncomplicated appendicitis. However, antibiotics have been shown to have some complications. Despite this being the case, the studies identified the potential of using antibiotics as a definitive treatment of uncomplicated AA in pediatrics. Further studies are required to evaluate the cost-effectiveness and recurrence of AA of this alternative treatment method.

## Introduction and background

Acute appendicitis (AA) is cited as the leading cause of surgical acute abdomen in pediatrics, and also the most frequent urgent surgical pathology in pediatrics worldwide. It majorly affects children aged two years and above, and adolescents, accounting for approximately 8% of pediatric emergency visits presenting abdominal pain [1]. Early diagnosis and treatment for AA are recommended to significantly reduce complications associated with appendicitis such as fecal peritonitis, formation of abscess, perforation, and sepsis [1].

For a long time, surgical appendectomy has been effectively used as the first-line treatment for AA. Appendectomy which is regarded as the standard treatment strategy for AA is associated with high efficiency and effectiveness, and low mortality. Other conservative management practices such as the use of antibiotics have been applied in the treatment of appendicitis but are still controversial. Some medical conditions may necessitate medical treatment, but the absence of clear evidence hesitate this decision.

Although the pediatric population was less affected by COVID-19 as compared to the adult population, the pandemic has significantly altered AA surgical protocols and management guidelines [2]. Percul et al. [3] also confirm that during the pandemic period, the number of visits to the emergency department due to AA significantly reduced, majorly due to reassessed management strategies. COVID-19 has had a significant impact on the surgical treatment strategies of AA in pediatrics, with many pediatric surgeons having to shift from upfront surgical appendectomy to conservative management involving the use of antibiotics as a treatment strategy. COVID-19 has overwhelmed most hospitals in the U.S. and across the world hence creating difficult access to health services and operating rooms for AA pediatric patients. Therefore, this study seeks to establish the COVID-19 influence on the decision of medical versus surgical pediatric AA exploiting the pandemic effect.

## Review

Materials and methods

**Figure 1:**
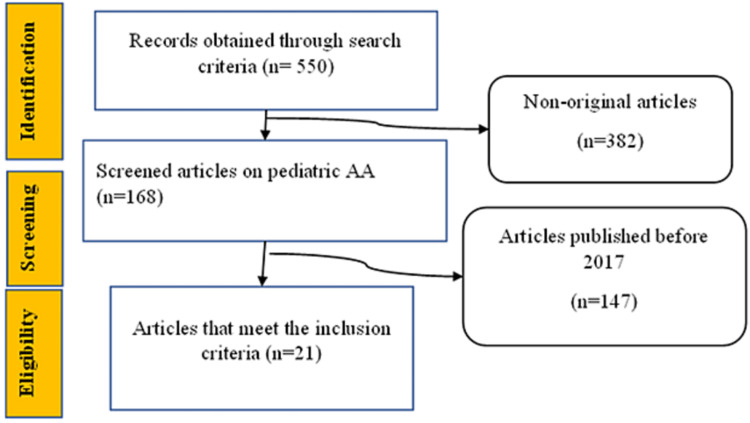
A diagram showing the selection and screening process

This is a meta-analysis study that compares the outcomes between appendectomy and conservative therapy in the management of AA in pediatrics. Results from various retrospective studies, prospective clinical controlled trials, correlational studies, and randomized clinical trials were analyzed (Figure 1). All studies including pediatric AA management during COVID-19 were included. The search selected to be less than five years utilized key medical words such as appendicitis, appendectomy, conservative management, antibiotics, appendicular perforation, appendicular abscess, appendicular gangrene, appendicular phlegmon, pediatrics, and Covid-19. Information and data on the topic were searched in the Cochrane Library (PubMed, CDSR, CENTRAL, DARE, Embase, Medline, and other medical databases), and peer-reviewed journals that are published not more than five years ago.

Titles and abstracts were independently screened by two reviewers against the set eligibility criteria. After screening the title and abstract of every study, full-text screening was conducted to qualify the papers. Any disagreement between the two reviewers at any stage was resolved through the help of a third party or discussion. The meta-analysis included studies that covered surgical and conservative management of AA among children.

Outcomes

The primary outcome assessed in this meta-analysis is the effectiveness of the two approaches in the management of AA during the COVID-19 period.

Risk of bias assessment

The two reviewers independently conducted risk of bias assessment for all the studies using the AXIS tool for the cross-sectional study, the ROBINS-I for the retrospective studies, the Navigation Guide risk of bias checklist for observational studies, and the ROBIS tool for meta-analysis.

Data extraction

Relevant data were extracted from the studies using a data extraction form that was created on Microsoft excel. The data extracted include the name of the author and year of publication, the study design used, the sample size for each group, and outcome measures.

Statistical analysis

SPSS version 25 was used for all statistical analyses. Descriptive statistics were majorly used in presenting the data. The random effects model was used to pool data for dichotomous outcomes. The mantel-Haenszel model was used to compute risk ratios with 95% confidence intervals.

Results

**Figure 2:**
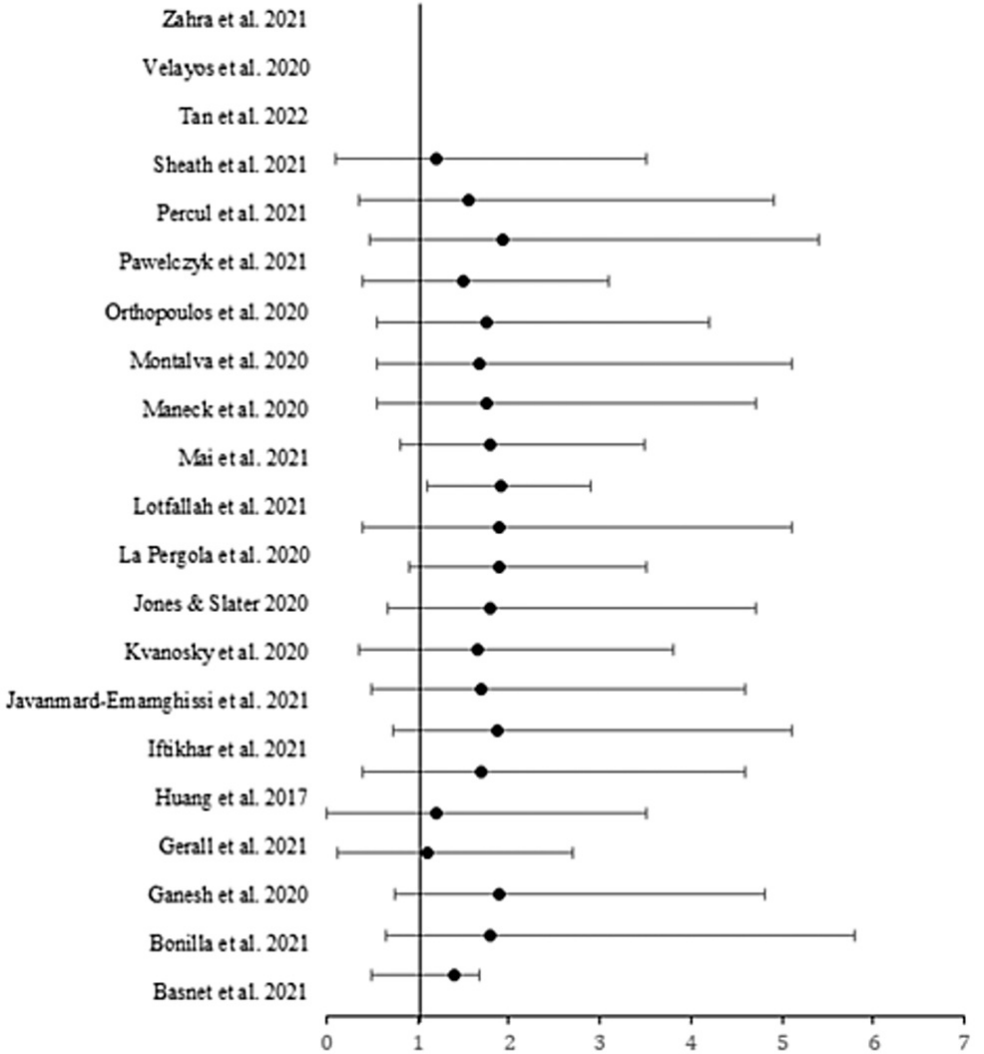
Forest plot showing risk ratios and confidence intervals [1-16,19-23]

The search yielded 21 articles that fully met the inclusion criteria. Articles that were published more than five years ago were excluded from the analysis. Also, articles that included studies on the adult population were excluded. Following the studies that were included in this review, the study population included children between the age of 10 to 18 years. All the studies included in this meta-analysis had relatively similar weights as shown by the graphical representation of the odds ratio the forest plot (Figure 2). All the studies utilized both conservative therapy and appendectomy. Conservative therapy involved the use of various antibiotics, which did not very much in most of the studies. Laparoscopic and open surgery were the major appendectomy procedures performed on pediatrics who were diagnosed with AA. Since the study focused on the COVID-19 period, all the articles included in the meta-analysis covered healthcare facilities that had similar COVID-19 protocols. The included studies report that most of the hospital departments were repurposed to handle COVID-19 patients which has had an impact on the presentation of other conditions.

Among all the included studies, only seven studies reported on COVID-19 positivity rates (Percul et al., Kvanosky et al., Orthopoulos et al., Gerall et al., Pawelczyk et al., La Pergola et al., Javanmard-Emamghissi et al.). COVID-19 positivity rate ranged between 1% and 10% with Javanmard-Emamghissi et al. recording the lowest positivity of 1% while Orthopoulos et al. and La Pergola et al. reported the highest rates of 8% and 10%, respectively.

Characteristics of studies selected

This study summarized the attributes of the 21 included studies (Table 1). A total of 5,137 individuals with AA were included, with 3,799 being assigned to the surgical group and 1,338 being assigned to the conservative group. The diagnosis of all suspected cases of AA was obtained from patient history, radiographic evidence, clinical signs, and laboratory tests. In many of the studies, ultrasonography or computed tomography confirmed the diagnosis. All the patients who received appendectomies had their diagnosis confirmed by pathologic results and there were no negative appendectomies. Cases of appendicitis declined by 70% to 80% from the pre-COVID period to the COVID-19 period.

**Table 1:** The characteristics of the selected studies

Author	Year	Study design	Groups	Sample size	Mean age (Years)
Lotfallah et al. [1]	2021	Retrospective study	Surgical	31	11
			Conservative	26	
Zahra et al. [2]	2021	Retrospective cohort study	Surgical Conservative	172 95	10
Percul et al. [3]	2021	Analytical retrospective comparative study	Surgical	88	11.5
			Basnet et al. [4]	2021		Descriptive cross-sectional study	Surgical	35	11
Conservative	44								
Bonilla et al. [5]	2021	Retrospective observational cohort study	Surgical	90	10
			Kvanosky et al. [6]	2020		Retrospective study	Surgical Conservative	30 25	12.4
Velayos et al. [7]	2020	Retrospective observational study	Surgical	66	10.7
Orthopoulos et al. [8]	2020	Retrospective review	Surgical	89	11
			Conservative	10	
Gerall et al. [9]	2021	Retrospective review	Surgical	20	13
			Conservative	12	
Maneck et al. [10]	2020	Retrospective study	Surgical	400	12.5
Montalva et al. [11]	2020	Retrospective cohort study	Surgical	108	11.1
Sheath et al. [12]	2021	Retrospective comparative study	Surgical	171	12
			Conservative	1	
Pawelczyk et al. [13]	2021	Retrospective review	Surgical	365	12
Jones & Slater [14]	2020	Case study	Conservative	1	13
Tan et al. [15]	2022	Prospective case-control study	Surgical Conservative	61 78	10
Ganesh et al. [16]	2020	Retrospective study	Surgical	82	14
[17], [18].			Conservative	14	
La Pergola et al. [19]	2020	Retrospective study	Surgical	86	10
Huang et al. [20]	2017	Meta-analysis	Surgical Conservative	236 168	13.5
Iftikhar et al. [21]	2021	Observational study	Surgical	42	13
			Conservative	16	
Mai et al. [22]	2021	Observational study	Surgical	72	11
			Conservative	17	
Javanmard-Emamghissi et al. [23]	2021	Prospective cohort study	Surgical Conservative	2018 1402	17

**Table 2:** Assessment of quality for observational studies

Study	Selection	Comparability	Outcome	Overall quality
Bonilla et al. 2021 [5]	4	3	4	Good
Iftikhar et al. 2021 [21]	3	4	4	Good
Mai et al. 2021 [22]	4	2	3	Good

From the analysis of the articles, three outcomes were recorded. The first one was the efficacy of the treatment method. The efficacy of the conservative group was evaluated as the resolution of symptoms among patients without necessitating surgery within two days of appendicitis recurrence within a month after initiating treatment. For the surgical group, efficacy was viewed as an operation without any negative appendectomy outcomes and/or reoperation. The effective treatment rate for conservative therapy was 77.5% (95% CI: 66.5%-88.5%) while that of appendectomy was 90.8% (95% CI: 82.8%-98.9%). In general, a significant reduction in treatment efficacy rate was observed in the conservative therapy while a significant improvement was observed in treatment management by appendectomy (Table 3). In the conservative group, complications referred to an abscess, gangrene, perforation, and/or complications after appendectomy. The complications reported in this study for the conservative group include AA and antibiotic-related complications. For the surgical group, complications referred to post-operative complications such as surgical site infection, ileus, or any other post-operative readmission. From the study analysis, a complication rate of 11.5% was registered with the conservative treatment method while a 3.1% complication rate was observed with the appendectomy treatment method. This indicates that there were significantly higher rates of complications recorded in the conservative therapy than in an appendectomy (Table 4). Generally, the results showed that the number of appendicitis cases with abscess formation, post-operative intestinal obstruction, perforation, and readmission was higher in the COVID-19 period. The last outcome considered was the length of stay (LOS) in a hospital after administering the treatment. LOS was reported in four studies. Analysis of the studies showed that for the conservative treatment, patients had a significantly longer LOS in the hospital than the patients treated by appendectomy (Table 5).

**Table 3:** Treatment effective rate

Study type	Use of antibiotics	Appendectomy
Retrospective studies	80%	97%
Prospective cohort studies	80%	96.4%
Observational studies	75%	88.5%
Meta-analysis studies	78%	89%

**Table 4:** Complication rate

Study type	Use of antibiotics	Appendectomy
Retrospective studies	9.9%	3.1%
Prospective cohort studies	10%	1.8%
Observational studies	11.5%	1.6%
Meta-analysis studies	10.4%	2.2%

**Table 5:** Increased Length of Stay (LOS) (Average Days) for the conservative and surgical groups

Study type	Average days	
	Use of antibiotics	Appendectomy
Retrospective studies	0.6	0.1
Prospective cohort studies	0.5	0.2
Observational studies	0.7	0.1
Meta-analysis studies	0.4	0.3

Discussion

All the articles included in this analysis had relatively similar odd ratios which resulted in them being similar weight in contributing towards this study (Figure 2). Although the issue of AA has been well studied among adults, there is a gap in explaining the safety and feasibility of conservative treatment versus surgical interventions among children. The current study compared primarily the two interventions, particularly during the COVID-19 period. Twenty-one studies with different study designs were included to compare the outcomes of surgical and conservative interventions on length of hospital stay, complications as well as the effectiveness of the interventions. 

The effect of COVID-19 on cases reported in hospitals

The results of the study showed that COVID-19 had a significant effect on cases of appendicitis in emergency rooms. The COVID-19 period fundamentally changed global healthcare systems which affected services such as appendicectomies [4-7]. In a study conducted by Percul et al. [3] to establish the impact of COVID-19 on the treatment and management of AA pediatric patients, findings indicate a decline in the appendicitis cases reported at the emergency department. The study reveals that during the COVID-19 pandemic, a decline of about 70%-80% in pediatric emergency department visits was reported, compared to the 2018 and 2019 pre-pandemic period [3]. This decline was a result of the shift in attention towards the overwhelming COVID-19 cases which occupied the majority of the hospitals' departments including emergency. The decline in PED visits was also associated with the new stay-at-home COVID-19 guidelines, further delaying seeking medical care. This resulted in delays in diagnosis and treatment whose consequences include an increase in complications due to appendicitis. Kvasnovsky et al. [6] report that during the COVID-19 period, wards of children's hospitals became condensed as both emergency and elective care across many pediatric specialties, and many of the wards were converted t adult wards to manage the pandemic. This probably affected patients with appendicitis. Furthermore, Orthopoulos et al. [8] demonstrated an increase in cases of complicated AA at the onset of COVID-19 due to the postponement of elective surgeries. The findings of the study can be explained by the encouragement of the public to avoid unnecessary presentations to health care centers.

 Similar findings are also echoed by Zahra et al. [2]. Study findings indicate a decrease in the number of patients treated for appendicitis during the pandemic period as opposed to the pre-pandemic period. The study also reports a significant increase in complications of appendicitis as a result of delayed presentation and late conservative treatment application. Findings of the study associate decreased hospital visits by appendicitis pediatric patients with the COVID-19 protocol of staying at home, patients fearing contracting COVID-19 during hospital visits, and the revised AA management guidelines which emphasize more on the conservative treatment method to minimize the surgical lists.

 Conservative approaches were adopted in the management of acute surgical conditions recognizing the risks of increased mortality and postoperative morbidity among COVID-19-infected individuals. Also, the restrictions that were associated with the pandemic affected the normal delivery of health care globally [5,9]. The onset of the COVID-19 pandemic in a way affected how patients with appendicitis receive care which necessitated more conservative approaches. In a study conducted by Maneck et al. [10] to evaluate the effect of the obligatory confinement COVID-19 restrictions during the lockdown on the appendectomies done, findings indicate a significant decrease in the number of appendectomies done in 2020 during the lockdown period, compared with the ones done in 2019 and 2018 pre-pandemic period (p < 0.001). This is majorly attributed to the medical confinement measures that were introduced to curb the spread of the Coronavirus. Elective operations, screening measures such as mammography, scheduled hospitalizations, and outpatient clinics were reduced during the lockdown period. However, this operation affected mainly the simple and uncomplicated AA. The management of complicated AA by appendectomy was not affected. Additionally, Gerall et al. [9] report that at the peak of the pandemic, many additional sources of delay affected both complicated and uncomplicated AA. With healthcare resources being focused on the management of COVID-19, surgical procedures for AA are reduced.

 On the contrary, Montalva et al. [11] indicate an increase of 70% of children treated with AA at the pediatric surgery department during the lockdown period. Although most of the hospitals were restructured to cater to COVID-19 patients leading to the cancelation of elective surgeries which were not urgent and the closure of some children's surgery centers, there was no significant change in the provision of surgical activities for pediatric surgical emergencies, including AA, in the pediatric tertiary care center. In this study, no significant difference in complications due to AA was observed during the pandemic and pre-pandemic periods [11].

 The results of this study showed the increased length of hospital stay. Sheath et al. and Pawelczyk et al. [12,13] report that the differences in the courses of treatment for AA consequently increased the length of hospital stay for patients. The study also notes that delayed admissions to healthcare centers, limited proper diagnosis during the early stages, and in consequence causing late appendectomy could explain the high percentages of complicated cases of AA reported among the pediatric population during the COVID-19 period. Additionally, many of the patients were forced to stay at home till very late due to fear of contracting COVID-19 which resulted in increased problems with AA.

Surgical versus conservative management of AA

Appendectomy is the surgical management of appendicitis while conservative management involves the use of antibiotics to treat AA [14]. Over decades, appendectomy has remained to be the standard and most applied treatment method for appendicitis [4,5,15]. However, several studies have confirmed that conservative management strategies of using antibiotics are safe and effective in the treatment of uncomplicated appendicitis. Lotfallah et al. [1] affirm this from their meta-analysis and systemic reviews which conclude that while appendectomy remains the golden standard treatment strategy for appendicitis, the use of antibiotics has proven to be a safe and effective alternative. This finding is also backed by other evidence-based research studies recently carried out in India, the USA, and Europe, which affirms the safety and efficacy of conservative management of appendicitis using oral or intravenous antibiotics in both children and adults [16,17].

The impact of the COVID-19 pandemic has led to a decline in surgical capacity due to the mobilization of surgical staff to medical wards and critical units to cater to the overwhelming cases of COVID-19. This was observed during the first wave of the pandemic in the UK where some AA pediatric patients who would have been treated surgically received conservative treatment [1]. Findings of another study done by Livingston [18] to evaluate the success of antibiotic treatment versus laparoscopic appendectomy for uncomplicated appendicitis in pediatrics indicate that antibiotic therapy demonstrated a high success rate of 73% for one year and 61% for five years. Basnet et al., Tan et al. and La Pergola et al. [4,15,19] also report the safety and effectiveness of conservative methods in the management of uncomplicated AA. The study reports that conservative approaches in pediatric populations allow for the avoidance of complications that may arise from the surgery itself or general anesthesia. According to Basnet et al. [4], complications brought by COVID-19 and the invasive nature of surgical methods promote conservative approaches as viable alternatives. However, according to a study done by Huang et al. [20], although the initial antibiotic therapy to AA pediatric patients was effective with minimal complications, a higher failure rate majorly due to the presence of appendicolith was observed in conservative therapy than in appendectomy therapy. Similarly, Iftikhar et al. [21] report contrary outcomes in which a very large percentage (72%) required an operation since the condition worsened after using non-operative treatment. Mai et al. [22] also reported that management of simple AA is highly likely to fail compared to initial appendicectomy.

In a study done by Javanmard-Emamghissi et al. [23] to establish the efficacy of the use of antibiotics as the first-line alternative to appendectomy in the treatment of AA in children, non-operative management (NOM) using antibiotics is also an effective first-line treatment method for AA in children. In a prospective study that involved the use of antibiotics for pediatric patients who were diagnosed with uncomplicated AA, findings indicate a success rate of 80% of the total cases. Fewer overall complications and shorter lengths of stay were reported with the conservative treatment method of using antibiotics. Another study done in Germany supports these findings. In a study done by Maneck et al. [10] to assess the influence of COVID-19 on the management of AA, findings indicate a significant reduction in the number of appendectomies, with a shift to practicing conservative therapy than appendectomy. During the COVID-19 lockdown period, uncomplicated AA was treated non-operatively, with a success rate of about 82%. This confirms the findings of our research that the use of antibiotics can be adopted as an alternative first-line treatment method in the management of uncomplicated AA in pediatrics. Kvasnovsky et al. [6] could apply NOM to nearly half of their patients presented with acute with encouraging results of less surgical intervention and shortened hospital stay. The need for NOM in the pediatric population could be useful in cases like AA with pediatric hematological malignancies and autoimmune diseases undergoing chemotherapy to reduce morbidity and mortality during the treatment course. As well as it will help patients' parents to be able to make an informed decision between antibiotics treatment and appendectomy [24].

Limitations

This meta-analysis has some limitations, there were no randomized control trials that fit the inclusion criteria. Nonetheless, the studies that were included in the analysis were of high quality per the tools used for risk of bias assessment.

## Conclusions

AA is the most frequent surgical emergency in pediatrics, affecting majorly children from two years of age to adolescents. Management strategies such as surgical and conservative therapy have been utilized. Surgical therapy involves both open and laparoscopic appendectomy while conservative therapy involves the use of antibiotics. Although appendectomy remains the standard treatment method, the use of antibiotics has been demonstrated to be safe and effective in the treatment of uncomplicated appendicitis during the COVID-19 pandemic. It's also useful for those under active chemotherapy for malignancies. Therefore, antibiotics can be used as an alternative first-line treatment for the treatment of uncomplicated AA in pediatrics particularly in times when accessing surgical treatment is difficult for instance during the COVID-19 period. However, due to the higher complication rates reported in studies, this approach may not be definitively used alone since complications necessitate surgical procedures. Further studies are required to evaluate the cost-effectiveness and recurrence of AA of this alternative treatment method.

Implications for future research

The findings of this meta-analysis show the potential of the use of conservative methods as a definitive treatment for uncomplicated AA despite the higher complication rates compared to surgical procedures. These findings, therefore, support the usage of antibiotics even though a lot of care needs to be taken. This can influence policies surrounding the management of AA, particularly among the pediatric population since it is non-invasive. However, there is very limited information on the cost implications of using conservative methods compared to surgical methods in the management of pediatric AA. Further research is needed in this area to evaluate cost implications and compare the findings against efficacy levels.
